# More Power with Flower for the Pupal Parasitoid *Trichopria drosophilae*: A Candidate for Biological Control of the Spotted Wing Drosophila

**DOI:** 10.3390/insects12070628

**Published:** 2021-07-10

**Authors:** Annette Herz, Eva Dingeldey, Camilla Englert

**Affiliations:** Julius Kühn Institute (JKI)—Federal Research Centre for Cultivated Plants, Institute for Biological Control, Heinrichstr. 243, 64287 Darmstadt, Germany; eva.dingeldey@gmail.com (E.D.); camillaenglert@gmx.de (C.E.)

**Keywords:** biological control, nutrition, flower resources, *Drosophila suzukii*

## Abstract

**Simple Summary:**

Parasitic wasps are important natural enemies of the spotted wing drosophila, an invasive fruit pest. Releases of mass reared wasps require the presence of all resources necessary to ensure their effectiveness in the crop system. We investigated the utility of floral resources to feed *Trichopria drosophilae*, one of the candidate species, in a laboratory study. Survival of males and females increased by three to four times when they had access to flowers of buckwheat or of two cultivars of sweet alyssum. The number of offspring produced was also much higher for flower-fed wasps. Given that almost a threefold increase in overall fitness of the wasps was observed, it is advisable to introduce flowering plants into the crop system to enhance their activity for biological control of the spotted wing drosophila. However, any unwanted advantages on the pest itself need to be carefully avoided.

**Abstract:**

Parasitoids are currently considered for biological control of the spotted wing drosophila (SWD) in berry crops. Releases of mass-reared parasitoids require the presence of all resources necessary to ensure their effectiveness in the crop system. The use of floral resources to feed *Trichopria drosophilae*, one of the candidate species, was investigated in a laboratory study. The life expectancy of males and females increased by three to four times when they had access to flowers of buckwheat or of two cultivars of sweet alyssum. Female realized lifetime fecundity increased from 27 offspring/female exposed to water only to 69 offspring/female exposed to buckwheat flowers. According to this almost threefold increase in parasitoid fitness, it is advisable to introduce flowering plants into the crop system, when parasitoid releases are carried out. Sweet alyssum offers the advantage of not growing too tall in combination with an extended blooming. However, adult SWD were also able to feed on flowers of both plants and survived for at least 27 days, much longer than starving flies. The introduction of flowering plants to promote natural enemies therefore requires further consideration of the risk–benefit balance under field conditions to prevent unintended reinforcement of this pest.

## 1. Introduction

Adults of many parasitoid Hymenoptera depend on regular access to sugar resources for nutrition [1]. Such resources may consist of flowers with accessible or extrafloral nectaries, plant saps or tree fluids, aphid honeydew, or any other sugar-containing material, e.g., from fruits. Provision with such sugar sources in agroecosystems is often essential for optimal parasitoid performance in biocontrol programs. Comprehensive research resulted in the general recommendation to introduce suitable flowering plants into the cropping system for the promotion of natural enemies [2,3].

The pupal parasitoid *Trichopria drosophilae* (Perkins) (Hymenoptera, Diapriidae) is currently under investigation for its use in the biological control of *Drosophila suzukii* (Matsumura) in Europe and other countries where this fruit-damaging species has invaded in recent years. Strategies to exploit control capacity of *T. drosophilae* can be seen in conservation biocontrol, use in augmentative releases in the crop system, or a mixed approach by inoculative releases and subsequent enhancement of the parasitoid population [4,5,6,7,8]. Regular inundative releases may be one promising option, especially in highly susceptible fruit crops which are attacked by *D. suzukii*, e.g., raspberries, cherries, and blueberries [7,9,10]. The intention is to suppress growing pest populations in a way that infestation of fruits is not too massive. Other essential measures, such as frequent picking, the regular removal of heavy infested fruits, and the prevention of access by protective nets around the crop, can then be more efficient in reducing economic damage. Especially during periods of low host density, maintaining or enhancing physical fitness of released parasitoids must be possible in order to enable their survival, mobility, and host searching efficacy. The need of *T. drosophilae* for sugar resources has been demonstrated in laboratory cultures where the parasitoids are typically fed with honey [11]. A recent study also discovered the suitability of flowers (buckwheat, cornflower) and blueberries (infested and uninfested) to prolong the lifespan of *T. drosophilae* [12]. Females of *T. drosophilae* do not perform host feeding [13,14], probably because the parasitoid larva develops as a endoparasitoid in the prepupa inside the fly puparium. Since this species is oligophagous and specialized on Drosophilidae, alternative hosts are likely to be scarce in a managed cropping system. Other resources must keep up performance of released parasitoids until sufficient host pupae are available.

Augmentative releases of *T. drosophilae* are especially promising in net-protected berry crops. In this situation, the crop is usually cultivated in a way that no other vegetation is allowed, resulting in a very simplified environment for the parasitoid. Sometimes, additional flowering plants are introduced for maintaining bumblebee colonies, which need to be established inside the net tunnels for crop pollination (blueberries, raspberries, and black berries). However, these wild bee-suited plants are often not usable for insects with unspecialized mouth parts including parasitoid wasps [15]. Instead, these wasps visit plants with open-disk flowers or extrafloral nectaries where the nectar is exposed and easily accessible for the wasps. Many studies confirmed the suitability of buckwheat (*Fagopyrum esculentum* Moench) and sweet alyssum (*Lobularia maritima* L. (Desv.) for various parasitoid species [16]. These annual plants are easy established by sowing. Moreover, sweet alyssum originates from the arid Macaronesian and Mediterranean regions, and is quite resistant against heat and drought. Consequently, it can grow as groundcover along the rows without affecting the development of the berry crop. Various cultivars of sweet alyssum exist due to their use as an ornamental plant, and show different colors and flower size. Due to breeding selection as an ornamental plant, they may have different flower characteristics and differ in their reward for flower visitors/pollinators as it was shown for other Brassicaceae [17].

The objective of the current laboratory study was to estimate the value of flowering plants, such as buckwheat and sweet alyssum, for increasing performance of the parasitoid *T. drosophilae* for releases in berry crops. We focused on the possible effects on reproducing males and females of *T. drosophilae* and compared their longevity as well as realized female fecundity at different diets. We also investigated the potential impacts of nutrition on sex ratio, developmental time, and size of the progeny. In a further experiment, these flowering plants were also offered to adult *D. suzukii* flies to test for unanticipated benefits for this target host.

## 2. Materials and Methods

### 2.1. Rearing of Insects and Preparation of Plants

The rearing culture of *D. suzukii* was collected from infested cherries in Baden-Wuerttemberg in 2013. Flies were held in wooden framed glass cages (50 cm × 35 cm × 41 cm) in a rearing room at 21 ± 2 °C and: 16:8 L(light):D(darkness) h photoperiod. Water and a mixture of brewer’s yeast and sugar (3:1) served as food for adult flies. Plastic cups (125 mL) containing a layer (2 cm high) of an artificial diet prepared from apple puree, yeast, wheat flour, sugar, and agar, were offered three times per week for oviposition and subsequent larval rearing. Development of larvae took place in the same rearing room. After pupation, some of the pupae were harvested for parasitoid rearing or the experiments. The rest was used to maintain the rearing stock of *D. suzukii*.

Our rearing strain “TD-WAR” of the parasitoid *T. drosophilae* was collected from pears infested by *Drosophila* sp. at Weil am Rhein, Baden-Wuerttemberg, Germany in the year 2015 [18,19]. The first generations were reared on *D. melanogaster* Meigen, but since 2017, they were reared exclusively on *D. suzukii* as host. About 30 to 50 adult parasitoids were kept in transparent plastic cylinders (12 cm diameter × 20 cm height) covered with glass petri dishes (13.5 cm diameter × 2 cm height). The wasps were provided with water and honey as food and with one- or two-day-old *D. suzukii* pupae as hosts. The rearing took place in a room conditioned at 23 ± 2 °C and 16:8 L:D. Pupae were replaced twice per week and exposed pupae were subsequently transferred to a climatic chamber (23 ± 0.5 °C, 70% relative humidity (RH), 16:8 L:D) until parasitoid emergence after approximately three weeks. The selected plant species *F. esculentum* (cultivar ‘Lileja’) and two cultivars of *L. maritima* (cultivar ‘Benthamii’ and cultivar ‘Tiny Tim’) were sown weekly into pots and cultivated under standard greenhouse conditions at 23 °C and long-day conditions. Daily watering and weekly fertilization supported fast and healthy growth and rapid flower regeneration. The flowering period of the plants lasted about four weeks in the greenhouse.

### 2.2. Evaluation of Floral Diet on Trichopria drosophilae Performance

Transparent plastic vials (‘Pint-sized Insect Pot’, 11 cm high, 12 cm diameter, volume 400 mL, Mega View Science Co., Taiwan) were all equipped with a 10 mL plastic vessel containing water and a cotton roll as a dispenser (water treatment, negative control “NC”). Depending on the treatment, a second vessel (medical cup) containing an inflorescence (treatment “FE”: *F. esculentum*, treatment “LBT”: *L. maritima* ‘Benthamii’, treatment “LTT”: *L. maritima* ‘Tiny Tim’) or a piece of parafilm with thin layers of honey (honey treatment, positive control “PC”) was added. One freshly emerged (<24 h old) pair of *T. drosophilae* was transferred into each experimental vial. The vial was sealed with a lid of metal gaze and positioned upside down to prevent the insects from being trapped in the meshes. In the following days, freshly emerged wasps were taken from the rearing and randomly assigned to the five treatments until there were 30 pairs for each treatment within three weeks. The flowers were changed every other day, while water and honey were changed once a week. In each of the flower treatments, three inflorescences of different developmental stages (fully opened, partially opened, and still closed buds) with about 15–20 flowers each were placed in the single vial. This arrangement ensured the provision of fresh flowers during the complete exposure period of two to three days, as confirmed in a preliminary test. Experimental vials were transferred to climatic chambers (Percival I-36VLC8) and held at 23 ± 0.5 °C, 70% RH, and 16:8 L:D. Survival of wasps was checked on each working day until death. Furthermore, ten one day old pupae of *D. suzukii* were added to each pair every second to third day. After exposure, pupae were further incubated in small plastic vessels in a climatic chamber at 23 ± 0.5 °C, 70% RH, 16:8 L:D, until progeny emerged. The day of the first appearance of males and females of each parasitism batch was noted to calculate developmental time, and emerged wasps were removed and killed by freezing. Male and female emerged wasps, flies and non-eclosed pupae were counted from each pupae sample to determine rate of parasitism. Twenty females were then randomly selected from the progeny of each parent and their size was estimated by measuring the tibia length of the left hind leg, according to [13].

### 2.3. Evaluation of Floral Diet on Drosophila suzukii

A simpler experiment assessed whether *D. suzukii* flies also feed on flowers of the target plants to prolong their lifespan. Freshly emerged flies were randomly distributed to four treatments: water only (negative control, “NC”), water & buckwheat flowers (“FE”), water & *L. maritima* ‘Benthamii’ flowers (“LBT”), and water & mixture (3:1) of brewer’s yeast and sugar (positive control, “PC”). The set-up of experimental vials and incubation conditions were identical to those described for parasitoids. Ten pairs were used for each treatment. A petri dish (diameter 3 cm) containing a simple medium (water + 2.5% agar without any sugar source) was offered twice per week for 24 h for oviposition. The acceptance of this medium for oviposition was checked previously in the *D. suzukii* rearing cages. On the following day, the oviposition medium was removed, and eggs were counted under the stereomicroscope. Survival of the flies was monitored on each working day for 27 days unless death occurred earlier. In addition, the length of the proboscis of *D. suzukii* was measured in ten males and ten females to assess their ability to reach nectaries of the flowers.

### 2.4. Data Analysis

Survival of *T. drosophilae* males and females was observed until death. Censoring occurred only for four wasps due to their escape from the experiment, whereas all *D. suzukii* that survived until day 27 were censored. Kaplan–Meier survival analysis was performed separately for male and female survival, followed by a post-hoc Log-rank test with Bonferroni corrections to compare survival rates between treatments. The number of offspring produced by wasps was used as an estimate for the number of originally parasitized hosts. Non-eclosed pupae accounted to 20% on average, thus being in the same range of *D. suzukii* rearing conditions, and were not considered in the analysis. The total number of offspring/female for wasps or total number of eggs/female for flies was compared between different diets in general linear models (GLM), assuming negative binomial data distribution. Parasitization performance of *T. drosophilae* over time was examined in general linear mixed models (GLMM) to test for the effect of diet and parasitism period (fixed factors) and individual replicate (random factor) on the number of offspring/female. The parasitism period was considered as an estimate for the age of females in days, and one interval between each change of host pupae referred to 2.3 days of female lifetime on average in the experiment. Effects of diet on developmental time of males and females and size or proportion of females of the F1-generation was examined as ANOVA (in case of normal error distribution) or GLM (binomial data distribution). Data were displayed as arithmetic mean ± standard error (SE). Depending on statistical testing, likelihood ratio test (LRT)-values, F-values, *Χ*^2^-values, z-values, degrees of freedom (d.f.), and *p*-values were calculated. Marginal means were pairwise compared by Tukey HSD testing. Statistical software used was R version 4.0.3—“Bunny-Wunnies Freak Out” [20]. Life table parameters were calculated following the description of [21] using the R Script developed by [22].

## 3. Results

### 3.1. Survival of Trichopria drosophilae at Different Diets

**Figure 1:**
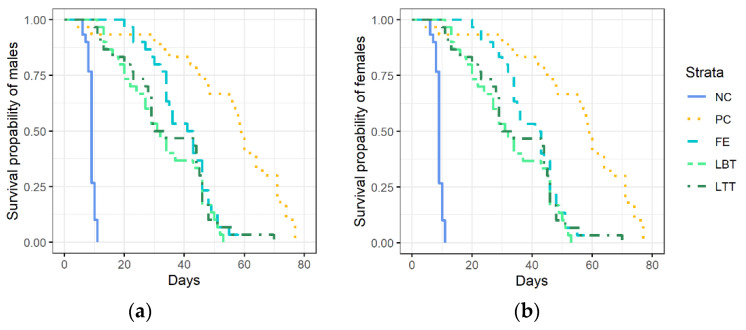
Survival of (**a**) male and (**b**) female *Trichopria drosophilae* when provided with different diets (NC = water, negative control, PC = honey, positive control; flowers of FE = *Fagopyrum esculentum*, LBT = *Lobularia maritima* ‘Benthamii’, LTT = *L. maritima* ‘Tiny Tim’). N = 30 wasps per treatment (=Strata).

The life expectancy of males and females of *T. drosophilae* depended significantly on the diet offered. (Figure 1, males: *Χ*^2^ = 241, d.f. = 4, *p* < 0.00001, females: *Χ^2^* = 213, d.f. = 4, *p* < 0.00001). Wasps survived less than 10 days when they were not offered sugary food sources (treatment NC: mean survival ± SE: males: 9.3 ± 0.2 days, females: 8.9 ± 0.2 days). When provided with honey, both sexes lived the longest time (treatment PC: males: 48.9 ± 1.6 days, females: 49.8 ± 2.6 days). When provided with flowers (FE, LBT, LTT), survival time increased three to fourfold compared to negative control (males: FE: 41.8 ± 1.5 days, LBT: 38.6 ± 1.9 days, LTT: 35.7 ± 2.4 days; females: FE: 39.5 ± 1.8 days, LBT: 32.6 ± 2.4 days, LTT: 34.2 ± 2.5 days). There were no significant differences between flower diets, but between flower diets and negative or positive control (Log-rank test, Bonferroni correction, *p* < 0.0001 in all comparisons).

### 3.2. Parasitism Performance of Trichopria drosophilae at Different Diets

**Figure 2:**
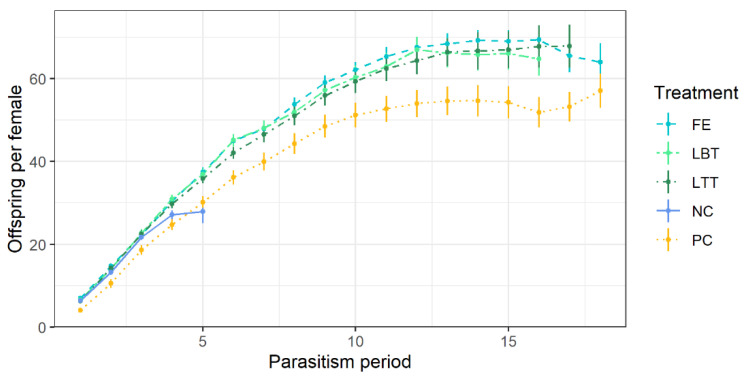
Cumulative number of offspring produced by female *Trichopria drosophilae* when provided with different diets (NC = water, negative control, PC = honey, positive control; flowers of FE = *Fagopyrum esculentum*, LBT = *Lobularia maritima* ‘Benthamii’, LTT = *L. maritima* ‘Tiny Tim’) and ten hosts (pupae of *Drosophila suzukii*) for each parasitism period. N = 30 wasps per treatment. Parasitism period refers to 2.3 days on average. Solid dots with lines display mean ± SE.

Diet significantly influenced total realized fecundity of females (GLM, formula = parasitized hosts ~ diet, LRT = 107.83, d.f. = 4, *p* < 0.0001). Females that fed on flowers produced more offspring than those that fed on honey or those that were starving (Figure 2). The average total of offspring accounted to 69.2 ± 4.4 a (FE), 61.5 ± 3.9 ab (LTT), 56.6 ± 3.7 ab (LBT), 53.1 ± 3.5 b (PC), and 27.1 ± 1.9 c (NC) offspring/female (different letters mark significance, post-hoc multiple comparison, Tukey HSD, *p* < 0.05)). Successful offspring production stopped at parasitism time 18 (= 41 days of female age) in all treatments (Figure 2).

**Figure 3:**
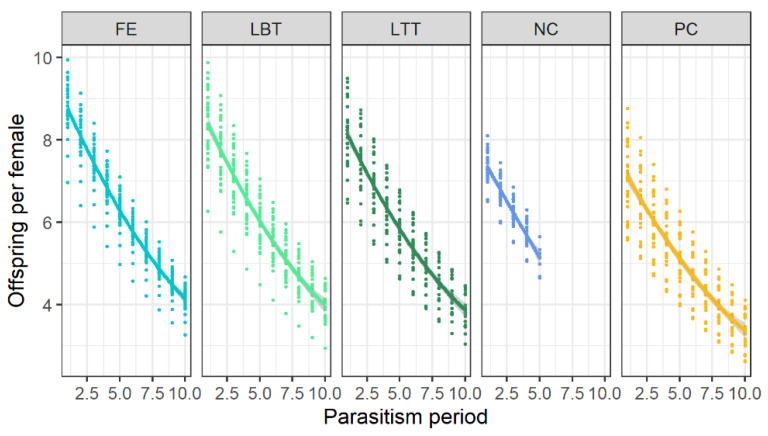
Offspring produced per parasitoid female/period up to the tenth parasitism period (=23 days of female age). Lines were drawn after fitting GLMM with negative binomial errors to the data of 30 females of *Trichopria drosophilae* per diet (NC = water, negative control, PC = honey, positive control; flowers of FE = *Fagopyrum esculentum*, LBT = *Lobularia maritima* ‘Benthamii’, LTT = *L. maritima* ‘Tiny Tim’). Parasitism period refers to 2.3 days on average.

Parasitism rates (offspring produced) changed over time in all treatments (GLMM, formula = offspring ~ diet + parasitism period + (1|ID Female); fixed effects: diet: LRT = 12.77, *p* = 0.012, parasitism period: LRT = 150.08, *p* < 0.0001). Parasitism rates were always higher when flowers were provided (Figure 3). All starving females died until parasitism period 5 (= after approximately 11 days of female age), nevertheless offspring production in this treatment had always been higher than in the honey treatment. In all diet treatments, offspring production steadily declined from period 2.0 (= 5 days of female age) onwards and, after period 10 (= 23 days of female age), less than four hosts/period were successfully parasitized (Figure 3).

### 3.3. Effects on F1-Progeny of Trichopria drosophilae at Different Diets

Males always developed faster than females (males: 19.6 ± 0.1 days, females: 20.4 ± 0.1 days, z.ratio: 11.429, *p* < 0.0001). Diet treatments of parental parasitoids had an effect on the developmental time of males, which lasted on average a half day longer when mothers had been fed with honey (ANOVA: F (d.f.: 4, 824) = 3.717, d.f. = 4, *p* = 0.0052). No effect of parental nutrition on the development of daughters was found (ANOVA: F (d.f.: 4, 770) = 1.079, *p* = 0.3654). The size of F1-females (estimated as hind tibia length) did not differ between diet treatments of parent parasitoids (hind tibia length at NC: 532 ± 6.1 µm, PC: 555 ± 5.9 µm, FE: 543 ± 5.3 µm, LBT: 546 ± 4.9 µm, LTT: 542 ± 6.5 µm; ANOVA: F (d.f.: 4, 105) = 1.937, *p* = 0.1097).

**Figure 4:**
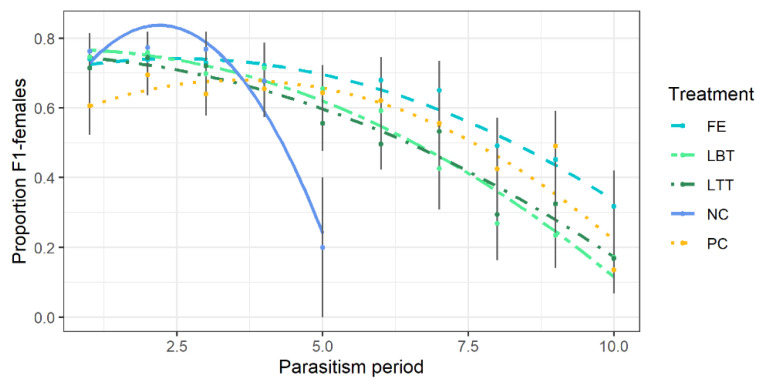
Proportion of females in offspring of *Trichopria drosophilae* fed with various diets (NC = water, negative control, PC = honey, positive control; flowers of FE = *Fagopyrum esculentum*, LBT = *Lobularia maritima* ‘Benthamii’, LTT = *L. maritima* ‘Tiny Tim’) up to the tenth parasitism period (= 23 days of female age). Solid dots display mean ± SE (grey lines). Parasitism period refers to 2.3 days on average.

The sex ratio (proportion of females) of the offspring produced until the tenth parasitism period declined with increasing age of the females of the parent generation (Figure 4). However, estimated marginal means of fitted GLMM (binomial data) were not significant from each other (NC: 0.74 ± 0.07 female proportion, PC: 0.68 ± 0.07 female proportion, FE: 0.62 ± 0.07 female proportion, LBT: 0.64 ± 0.08 female proportion, LTT: 0.56 ± 0.07 female proportion). Interaction of diet and the parasitism period significantly affected sex ratio over time (LRT = 597.47, d.f. = 5, *p* < 0.0001) due to different slope and intercept in NC and PC treatment in comparison to the flower diets (Figure 4). Female proportion was higher than 0.5 over a longer period in the treatments with FE and PC.

### 3.4. Life Table Parameters of Trichopria drosophilae at Different Diets

Demographic parameters according to [21] varied in the cohorts of *T. drosophilae* subjected to different diets (Table 1). Only descriptive statistics are possible, since no repetition of the whole experiment was performed. Nevertheless, the provision of flowers (buckwheat, sweet alyssum) resulted in the highest values of intrinsic rates of increase *r*, highest net reproduction *R_0_*, and lowest doubling times *DT*, indicating the best reproductive performance of the parasitoid by feeding on flower diets.

### 3.5. Effects of Different Diets on Drosophila suzukii Performance

**Figure 5:**
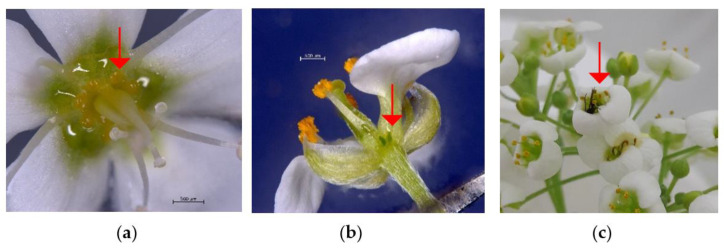
(**a**) Habitus of buckwheat flower with yellow-coloured nectaries (arrow), scale bar = 500 µm, (**b**) Habitus of alyssum flower with green nectaries (arrow), scale bar = 200 µm, (**c**) *T. drosophilae*-male (arrow) crawling into the alyssum flower.

Proboscis length of female *D. suzukii* accounted to 1082 ± 72 µm and was significantly longer than that of males with 910 ± 42 µm (ANOVA, F (d.f.: 1,19) = 42.074, *p* < 0.001). The proboscis length allowed access to open flowers of buckwheat, and obviously also to those of sweet alyssum (Figure 5a,b). Survival of *D. suzukii* males and females greatly increased by all floral diets and the yeast–sugar diet, in comparison to the water-fed flies (NC: males: 3.6 ± 0.25 days, females: 4 ± 0 days; PC: males: 27 ± 0 days, females: 27 ± 0 days, FE: males: 27 ± 0 days, females: 23.6 ± 2.31 days, LBT: males: 26.8 ± 0.19 days, females: 27 ± 0 days). All flies in the negative control died within four days, whereas more than 70% of the flies survived in the other treatments until the end of the experiment on day 27. All surviving flies were censored in the Kaplan–Meier analysis which indicated a significant effect of diet on survival (males: *Χ^2^* = 42.6, d.f. = 3, *p* < 0.0001, females: *Χ*^2^ = 35.4, d.f. = 3, *p* < 0.0001). Females did not readily oviposit into the provided medium. In the negative control, no single female oviposited, whereas 80% of females produced and laid eggs in the positive control, 60% in the FE treatment, and 20% in the LBT treatment. Diet had a significant effect on the total number of laid eggs (negative binomial GLM, LRT = 39.16, d.f. = 2, *p* < 0.0001). The number of eggs/female in the positive control was significantly higher than in the floral diet treatments (PC: 6.8 ± 1.8 a eggs/female, BW: 0.8 ± 0.4 b eggs/female, LBT: 0.3 ± 0.2 b eggs/female, different letters mark significance, post-hoc multiple comparison, Tukey HSD, *p* < 0.001).

## 4. Discussion

When provided with flowers or honey, reproducing females and males of *T. drosophilae* lived three to four times longer compared to wasps that had no opportunity to consume sugary nutrients. In addition, it has recently been shown that a mixture of buckwheat and cornflower (*Centaurea cyanus* L.) flowers or blueberries can also extend lifespan of host-deprived *T. drosophilae* fourfold [12]. We did not include parasitoids that had no hosts in our study design, although they are known to live even longer than those with hosts [12,23]. In any case, these findings are in agreement with many other studies on the nutritional ecology of parasitoids [1,16,24] and underline the need for flowering plants or other sugar sources in the habitat of *T. drosophilae*. In our study, while honey-fed wasps had the longest lifespan of more than 60 days (median), both flowering plants under examination guaranteed sufficient female survival to reach the highest realized lifetime fecundity, close to the estimated total egg load of this species (around 73 eggs/female according to [13]). In addition, flower-fed females produced the highest number of offspring per parasitism period from the beginning to the end of the reproduction time at about 40 days. Honey-fed females survived significantly longer, but their ability to parasitize also ended at day 40 (parasitism period 18). Therefore, it is likely that the floral rewards contain important nutrients that support the parasitization activity of wasps more than pure honey or that they simply met requirements of the parasitoid better [13]. In addition to various sugars, flower nectar also contains proteins, secondary plant metabolites, vitamins and other substances, and often bacteria or yeasts [25,26]. Flowers of buckwheat and sweet alyssum also provide abundant pollen. This finding suggests that these components are better suited for egg maturation when they come from fresh flowers instead of honey. It is also conceivable that certain auxiliary substances for successful host parasitization, such as secretions or venoms, may be better supplied by a nutrition from the flowers. Accordingly, the provision of flowering plants would probably be more important for maintaining the parasitizing capacity of *T. drosophilae* than the presence of other sugar sources, e.g., from fruits or from aphid honeydew. Such differentiations need to be elucidated in subsequent studies. We observed wasps gaining access on sweet alyssum by crawling into the flower to consume nectar secretions (Figure 5c). Diapriidae were found among parasitoids visiting flowers in field studies [27], which suggests that this parasitoid family use floral nectar to maintain locomotion and other body functions.

Diet had no direct effect on offspring sex ratio, which was in the range reported for *T. drosophilae* females in a situation free of local mate competition at the beginning of the oviposition period [28]. Honey- or buckwheat-fed females produced more daughters over a longer period than those on the other diets, probably because males also survived longer in these treatments. Thus, at parasitism period 10 (age of 23 days), more than 80% of the males in the honey and buckwheat variants were still alive. This situation could have led to more opportunities for (repeated) mating of the parents, avoiding sperm depletion and resulting in more fertilized eggs. However, it was reported that *T. drosophilae* females do not exhibit multiple mating [29]. Even so, males may gain important benefits from the presence of a suitable food supply, such as floral nectar. This is especially important under natural conditions when they have to expend even more energy to maintain their ability to disperse and find mates, or to exhibit a particular, energy-consuming courtship behavior [24].

The diet had no direct effects on developmental time or offspring size. The observed development rate under the described conditions of our experiment (23 ± 0.5 °C, 70% RH and long day conditions) agrees well with other studies on different rearing strains of this species [11,23]. As expected, males developed about one day faster than females. This arrhenotoky is typical of parasitoid Hymenoptera. The lack of effects of parental diet on offspring size can be explained by the fact that for an idiobiont, such as *T. drosophilae*, host size and quality are more crucial [30,31], although not confirmed by [32]. In general, effects of food quality on the enrichment of eggs with nutrients cannot be excluded in parasitoids [24]. On the other hand, larval hatching of *T. drosophilae* already takes place in a few hours after the egg has been laid into the host (unpublished data). Therefore, the neonate larva of the parasitoid certainly receives all necessary nutrients for growth from the host pupa.

The calculated demographic parameters of our *T. drosophilae* of German origin were in a comparable range to that reported for populations from Italy [23], California, and South Korea [11] under the same abiotic conditions (23 °C, 65% RH, 16:8 -L:D). However, those cohorts supplied with flowers of buckwheat or sweet alyssum achieved the highest life table values (*r* > 0.13, *R_0_* > 32) in our experiments. Hence, suitable and attractive floral resources clearly enhance the reproductive performance of *T. drosophilae*.

Tailoring the introduction of flowering plants for natural enemies also required a careful integration into the general crop management. Flowering plants can compete for water and nutrients or even hinder the growth and development of the crop. The establishment of flowering plants must also be simple and inexpensive, preferably by sowing. Sweet alyssum stays in flowers for a very long time, especially after one or two cuts; this thus matches perfectly with the proposed release period of the parasitoid from early summer to late autumn during susceptible stages of target crops (early and late ripening raspberries and blueberries). This plant ideally fulfills all the requirements for a suitable insectary plant and was found to increase fitness of various parasitoid species [33,34,35,36,37,38,39,40]. Hoverflies or predatory bugs, which can be important for controlling major berry pests, such as aphids or thrips, also benefit from sweet alyssum [41,42,43], making the introduction of this plant as a floral resource in berry production particularly worthwhile. Floral scent of sweet alyssum can be attractive for some parasitic Hymenoptera [3,36,44,45,46]. If confirmed also for *T. drosophilae*, this attraction may open the possibility to manipulate the dispersal of the wasp in the habitat and facilitate the movement between flower and host resource. Growing sweet alyssum near to the crop may direct the parasitoid to lower parts of the berry plant and near the soil where the major portion of *D. suzukii* pupae is expected [47]. We included two different cultivars of *L. maritima* in our study (‘Benthamii’ and ‘Tiny Tim’), but no significant differences in their floral rewards for *T. drosophilae* performance appeared, making both cultivars appropriate.

Unfortunately, tested flowering plants prolonged the survival of the host, *D. suzukii*. Extended lifespan of flies was also observed when they were exposed to cherry or blueberry blossoms [48]. We expected a similar effect for the buckwheat treatment, as its open flower structure abundantly offers nectar and as nectaries are easily accessible. In contrast, nectar glands of sweet alyssum were more hidden and less nectar was secreted (unpublished data). Nonetheless, the proboscis of *D. suzukii* was sufficiently long to reach the nectaries, and the flies may have even managed to crawl into the flower in a similar way to *T. drosophilae*. On the other hand, the egg-laying capacity of *D. suzukii* was not supported by flower diet alone. Egg production in drosophilid flies requires food rich in protein. Although nectar contains small amounts of protein, or may be colonized by bacteria and yeasts [25], the quantity is probably not sufficient. Pollen consumption and use for egg production, as known e.g., for hoverflies, has not been reported for Drosophilidae, with the exception of *D. flavohirta* Malloch. This species is able to feed on pollen by some kind of “external digestion” [49].

A further benefit/risk analysis of the enhancement of parasitoids or flies by floral resources centers around the need for studies under semi-field and field conditions. The fly does not have to visit (other) flowers in the berry crop system, since sufficient nutritional resources are available on the fruits (or the berry plant itself) and therefore the additional presence of the flowering plants does not matter. However, for the parasitoid, efforts for food foraging also need to be evaluated as well as the use of other sugar resources, e.g., from ripening fruits and fruit exudates (as found for *Diachasmimorpha longicaudata* (Ashmead) (Hymenoptera: Braconidae), a parasitoid of trephritid fruit flies [50], or larval parasitoids of Drosophilidae [51]). In a recent study [12], the suitability of blueberries (uninfested or infested by *D. suzukii*) was confirmed to enhance the lifespan of *T. drosophilae*. Further research needs to compare different available sugar sources for *T. drosophilae* in the particular cropping system. This will allow estimating the actual need for additional floral resources for the targeted promotion of important natural enemies in berry production.

## Figures and Tables

**Table 1:** Demographic parameters of *Trichopria drosophilae* held at 23 ± 0.5 °C, 70% RH, long day conditions and fed with various diets (NC = water, negative control, PC = honey, positive control; flowers of FE = *Fagopyrum esculentum*, LBT = *Lobularia maritima* ‘Benthamii’, LTT = *L. maritima* ‘Tiny Tim’) as adult females. Time unit of *R*_0_, *T* and *r* is [day]. N = 30 wasps per treatment.

*Demographic parameters*	NC	PC	FE	LBT	LTT
Net reproduction rate *R_0_*	18.0	29.3	42.8	37.5	32.3
Mean generation time *T*	24.5	20.1	30.5	28.9	28.6
Doubling time *DT*	5.8	5.8	5.2	5.2	5.3
Intrinsic rate of increase *r*	0.119	0.119	0.134	0.133	0.129

## Data Availability

Data are available from the article and from authors on request.
